# High-end normal adrenocorticotropic hormone and cortisol levels are associated with specific cardiovascular risk factors in pediatric obesity: a cross-sectional study

**DOI:** 10.1186/1741-7015-11-44

**Published:** 2013-02-20

**Authors:** Flavia Prodam, Roberta Ricotti, Valentina Agarla, Silvia Parlamento, Giulia Genoni, Caterina Balossini, Gillian Elisabeth Walker, Gianluca Aimaretti, Gianni Bona, Simonetta Bellone

**Affiliations:** 1 SCDU of Pediatrics, Department of Health Sciences, Università del Piemonte Orientale 'A. Avogadro', Via Solaroli 17, Novara, 28100, Italy; 2 Endocrinology, Department of Translational Medicine, Università del Piemonte Orientale 'A. Avogadro', Via Solaroli 17, Novara, 28100, Italy; 3 Interdisciplinary Center for Obesity Study (ICOS), Università del Piemonte Orientale 'A. Avogadro', Via Solaroli 17, Novara, 28100, Italy

**Keywords:** ACTH, cardiovascular risk, cortisol, glucose, hypertension, lipids, obesity, pediatric

## Abstract

**Background:**

The hypothalamic-pituitary-adrenal (HPA) axis, and in particular cortisol, has been reported to be involved in obesity-associated metabolic disturbances in adults and in selected populations of adolescents. The aim of this study was to investigate the association between morning adrenocorticotropic hormone (ACTH) and cortisol levels and cardiovascular risk factors in overweight or obese Caucasian children and adolescents.

**Methods:**

This cross-sectional study of 450 obese children and adolescents (aged 4 to 18 years) was performed in a tertiary referral center. ACTH, cortisol, cardiovascular risk factors (fasting and post-challenge glucose, high-density lipoprotein (HDL)-cholesterol, low-density lipoprotein (LDL)-cholesterol, triglycerides, and hypertension) and insulin resistance were evaluated. All analyses were corrected for confounding factors (sex, age, puberty, body mass index), and odds ratios were determined.

**Results:**

ACTH and cortisol levels were positively associated with systolic and diastolic blood pressure, triglycerides, fasting glucose and insulin resistance. Cortisol, but not ACTH, was also positively associated with LDL-cholesterol. When adjusted for confounding factors, an association between ACTH and 2 h post-oral glucose tolerance test glucose was revealed. After stratification according to cardiovascular risk factors and adjustment for possible confounding factors, ACTH levels were significantly higher in subjects with triglycerides ≥90th percentile (*P *<0.02) and impaired fasting glucose or glucose tolerance (*P *<0.001). Higher cortisol levels were found in subjects with blood pressure ≥95th percentile and LDL-cholesterol ≥90th percentile. Overall, the highest tertiles of ACTH (>5.92 pmol/l) and cortisol (>383.5 nmol/l) although within the normal range were associated with increases in cardiovascular risk factors in this population.

**Conclusions:**

In obese children and adolescents, high morning ACTH and cortisol levels are associated with cardiovascular risk factors. High ACTH levels are associated with high triglyceride levels and hyperglycemia, while high cortisol is associated with hypertension and high LDL-cholesterol. These specific relationships suggest complex mechanisms through which the HPA axis may contribute to metabolic impairments in obesity, and merit further investigations.

## Background

The prevalence of obesity in children and adolescents has increased over several decades in many countries [1,2]. This phenomenon has been accompanied by an increased incidence of type 2 diabetes and metabolic syndrome (MetS), which includes dyslipidemia and hypertension [3].

Cortisol has been reported to have a role in obesity, hypertension, and the altered glucose and lipid profile in Cushing's syndrome, and some studies have suggested that moderately increased morning fasting cortisol may be associated with the presence of cardiovascular risk factors in adults [4-6]. Abnormalities in the central regulation of the hypothalamic-pituitary-adrenal (HPA) axis due to stress may lead to a mild hypercortisolism in adults with obesity and MetS [7,8]. Reinher and Andler found significant associations between the degree of cortisolemia and fasting insulin levels in obese children, and levels of both hormones decreased following weight loss. These findings suggested that, in children, there are similar mechanisms to those reported in adults, and that higher cortisol levels are first a consequence rather than a cause of comorbidities in obesity [9]. A study in adolescents found racial/ethnic differences in daily cortisol secretion [10], but any studies in children have been limited to select or small populations. Two recent studies in overweight Latino youths with a family history of type 2 diabetes, confirmed higher fasting cortisol levels in those with lower insulin sensitivity [11] or MetS, and an association with hypertension and high glucose levels [12]. However, a study in a small group of prepubertal children showed higher morning plasma cortisol levels in those with higher total cholesterol and triglycerides [13]. Although cortisol is associated with metabolic alterations, it appears that adrenocorticotropic hormone (ACTH) may directly contribute to comorbidities in obesity. It has been shown *in vitro *that ACTH interacts with adipocytes, promotes insulin resistance and is proinflammatory [14]. To date, however, the role of ACTH has not been determined in obese children.

This study recruited a large cohort of overweight and obese pediatric subjects to determine the following: (1) to establish whether an association between cardiovascular risk factors and morning cortisol levels is present in obese Caucasian children and adolescents; (2) to evaluate whether ACTH is associated with cardiovascular risk factors in this population; and (3) to establish whether ACTH and cortisol levels are higher in those with specific cardiovascular risk factors.

## Methods

### Study design and population

This was a cross-sectional study. Study quality was assessed using the checklist of STROBE (for 'STrengthening the Reporting of OBservational studies in Epidemiology'; Additional file 1). We consecutively recruited 450 children and adolescents, aged 4 to 18 years, referred to the Pediatric Endocrine Service of our Hospital from January 2008 to October 2011 for obesity. The Hospital covers an area of North-East Piedmont with a population of approximately 500,000. The sampling rate was based on the age structure of the community and of the general pediatric population referred to the Service. Subjects were eligible if they were generally healthy, overweight or obese and not on a weight-loss diet (no engagement in any program to lose weight before the enrollment). Exclusion criteria were the known presence of diabetes or high blood pressure (BP), the use of drugs which influence glucose or lipid metabolism, specific causes of endocrine or genetic obesity, low birth weight, distress during blood sampling or a difficult phlebotomy (more than 5 minutes).

The protocol was conducted in accordance with the declaration of Helsinki and was approved by the Local Inter-Hospital Ethic Committee (Maggiore Hospital Ethical Committee). Informed consent was obtained from all parents prior to the evaluations after careful explanations were given to each patient.

### Anthropometric and biochemical measurements

All subjects underwent a clinical evaluation by a trained research team. Pubertal stages were determined by physical examination, using the criteria of Marshall and Tanner. Height was measured to the nearest 0.1 cm using a Harpenden stadiometer, and body weight with light clothing to the nearest 0.1 kg using a manual weighing scale. Body mass index (BMI) was calculated as body weight divided by squared height (kg/m^2^). The BMI standard deviation score (BMISDS) was calculated by the least median squares method [15]. Waist circumference was measured at the high point of the iliac crest around the abdomen and was recorded to the nearest 0.1 cm. Systolic BP (SBP) and diastolic BP (DBP) were measured three times at 2-minute intervals using a mercury sphygmomanometer with an appropriate cuff size after participants were seated quietly for at least 15 minutes, with their right arm supported at the level of the heart and feet flat on the floor, prior to other physical evaluations, and at least 30 minutes after blood sampling, using a standard mercury sphygmomanometer. Mean values were used for the analyses. Hypertension was determined if BP values recorded on enrollment day and on blood samples day are always elevated.

After a 12-hour overnight fast, children arrived at the clinical center at 7.30 AM and rested comfortably for half an hour prior to blood testing. At 8.00 AM, blood samples were taken for measurement of ACTH, cortisol, glucose, insulin, high-density lipoprotein (HDL)-cholesterol and triglycerides. ACTH and cortisol samples were drawn first. Subjects also underwent an oral glucose tolerance test (OGTT; 1.75 g of glucose solution per kg, maximum 75 g). Plasma samples were immediately separated and stored at-80°C. Children were screened for symptoms suggestive of Cushing's syndrome, and a 1 mg overnight dexamethasone suppression test and urinary free cortisol measurement were performed in case of suspicion. Signs of Cushing's syndrome were a low height percentile and high weight percentile as suggested by the guidelines of the Endocrine Society [16]. We also screened children for a height below that expected from parental height or a previous episode of severe hypertension. Children with positive screenings were excluded.

Insulin resistance was calculated using the homoeostasis model assessment of insulin resistance (HOMA-IR). Low-density lipoprotein (LDL)-cholesterol was calculated by the Friedwald formula. ACTH and cortisol levels were measured by an Immulite 2000 Medical System (Medical Systems S.p.A., Via Rio Torbido 40, Genova, Italy) (sensitivity: <12.0 pmol/l and <27.59 nmol/l, respectively). Other assays and formulas are as previously described [17].

### Definitions

Subjects were classified as overweight (BMI: 75th to 94th percentile) or obese (BMI: ≥95th percentile) according to Italian growth charts [15]. Children and adolescents underwent an evaluation for cardiovascular risk factors identified in the classification of MetS and by using cut-off values of the modified National Cholesterol Education Program-Adult Treatment Panel (NCEP-ATP) III criteria [18] as follows: (1) triglycerides ≥90th percentile for age and sex; (2) HDL-cholesterol ≤10th percentile for age and sex; and (3) impaired fasting glucose or glucose tolerance. High LDL-cholesterol was defined as ≥90th percentile for age and sex. Because of differences in the literature, hypertension was defined according to two specific cut-offs: (1) >95th percentile as suggested by the National High Blood Pressure Education Program (NHBPEP) Working Group of American Academy of Pediatrics (AAP) [19]; and (2) >90th percentile as suggested by definitions of pediatric MetS [18,20,21].

Triglyceride, LDL-cholesterol and HDL-cholesterol cut-off levels for age and sex were those used in the Lipid Research Clinic Pediatric Prevalence Study [22]. Impaired fasting glucose and impaired glucose tolerance were defined according to MetS and American Diabetes Association classifications as fasting plasma glucose of ≥5.6 to 6.9 nmol/l, and as 2-h post-OGTT glucose of ≥7.8 to 11.0 nmol/l, respectively [18]. Both SBP and DBP values were stratified according to percentiles of the NHBPEP Working Group [19].

### Statistical analysis

All data are expressed as mean ± standard deviation (SD), absolute values or percentages. A sample of 84 individuals has been estimated to be sufficient to demonstrate a difference of 27.59 µg/dl in cortisol with a SD of 2 with 90% power and a significance level of 95% using the Student's t test. Distributions of continuous variables were examined for skewness and were logarithmically transformed as appropriate. Correlation of ACTH and cortisol with continuous values of SBP, DBP, triglycerides, HDL-cholesterol and LDL-cholesterol, glucose, insulin, and HOMA-IR were examined using Pearson correlation coefficients. Partial correlation was used to correct for covariates. Analysis of covariance was used to determine differences in subjects with and without cardiovascular risk factors. Covariates were sex, age, pubertal stage, and BMI; BMISDS was used when the analysis was not contemporarily corrected for age. Analysis of covariance was also used to determine ACTH and cortisol differences among age and pubertal subgroups, with BMISDS (or BMI), HOMA-IR and sex as covariates. ACTH and cortisol were also categorized into tertiles. Multiple logistic regression was used to determine the association of tertiles of ACTH and cortisol with the odds ratio (OR, 95% CI) of each cardiovascular risk factor. Tertiles of ACTH and cortisol were included as independent variables, with the first tertile as the reference group. Statistical significance was assumed at *P *<0.05. The statistical analysis was performed with SPSS for Windows V.17.0 (SPSS Inc., Chicago, IL, USA).

## Results

### Anthropometric and metabolic phenotypes of all groups

The final dataset included 406 participants, aged 4 to 18 years (198 male, 208 female). A total of 29 subjects were excluded because they did not satisfy inclusion criteria (15 with difficult blood sampling, 5 with a low birth weight, 4 with hypothyroidism in thyroiditis, and 5 treated with glucocorticoids in the last 6 months). Other exclusions were two subjects diagnosed with late-onset congenital adrenal hyperplasia, eight with distress during BP monitoring, and five who refused the dexamethasone test. A total of 31 out of 406 subjects had the dexamethasone test and showed correct inhibition of cortisol levels (all <27.59 µg/dl), so Cushing's syndrome was excluded. We performed 24-h urine sampling for free cortisol measurement, but this was incomplete in 20 out of 31 patients.

**Figure 1:**
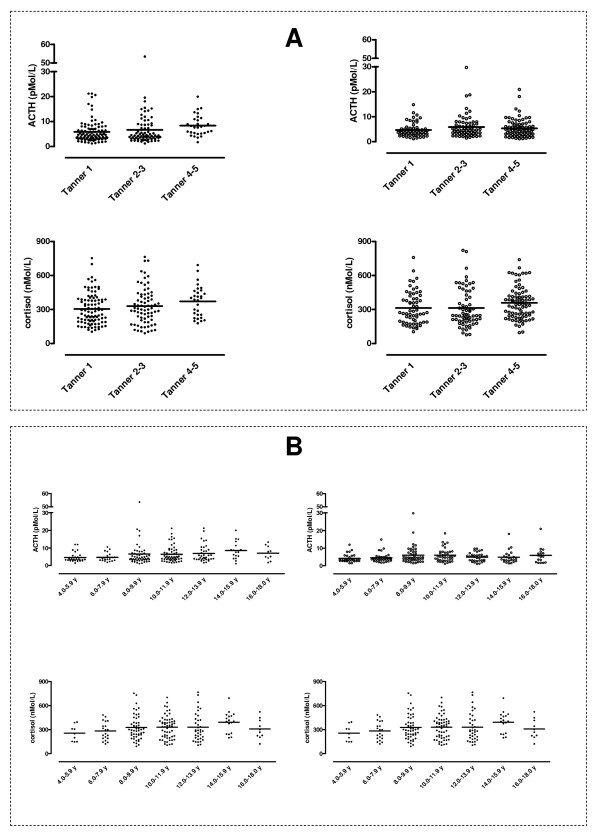
**Tanner-dependent (A) and age-dependent (B) adrenocorticotropic hormone (ACTH) (pmol/l) and cortisol (nmol/l) levels in 406 overweight and obese children and adolescents (filled circles, males, left panel; open circles, females, right panel)**. Cortisol levels were higher in the Tanner 4 to 5 stages than the Tanner 1 stage in the whole group (*P *<0.02*) and in females (*P *<0.02*). ACTH levels were higher in Tanner 4 to 5 stages (*P *<0.001*) and in 14.0 to 15.9 years (*P *<0.01*) in males than in females. Significance was maintained when the model included body mass index (BMI) (or BMI standard deviation score (BMISDS)) and homoeostasis model assessment of insulin resistance (HOMA-IR) as covariates. *Indicates significance of analysis of covariance (ANCOVA) analysis.

ACTH levels were higher in Tanner 4 to 5 stage participants (*P *<0.001) and in 14.0 to 15.9-year-olds (*P *<0.01) in males than in females, and this was also the case in the analysis corrected for BMISDS (or BMI) and HOMA-IR. Cortisol levels were higher in Tanner 4 to 5 stage participants than in Tanner 1 stage participants for the whole group (*P *<0.02), and in females (*P *<0.02); this was also the case in the corrected analysis for BMISDS and HOMA-IR (Figure 1).

A total of 97 (24.0%) subjects were overweight and 309 (76.0%) were obese. Of the 406 subjects included, 23 (5.7%) had impaired fasting glucose, 10 (2.5%) had impaired glucose tolerance and 5 (1.2%) had both. None were diabetic. A total of 91 (22.4%) subjects had triglycerides ≥90th percentile, 216 (53.2%) had HDL-cholesterol ≤10th percentile and 25 (6.2%) had LDL-cholesterol ≥90th percentile for age and sex. Hypertension according to NCEP-ATP criteria was diagnosed in 339 (83.4%) subjects and in 274 (67.4%), according to AAP criteria. Only 1 subject presented with all cardiovascular risk factors, while 63 (15.5%) had none. All clinical and biochemical characteristics are shown in Tables 1 and 2.

**Table 1:** Biochemical and clinical characteristics of subjects

	All	Tanner 1 to 2	Tanner 2 to 3	Tanner 4 to 5
Subjects	406	154	140	112
M	198	93	75	30
F	208	61	65	82
Age, years	10.7 ± 3.1	8.4 ± 2.3	10.4 ± 2.0	14.2 ± 2.0
M	10.6 ± 2.8	9.0 ± 2.2	11.1 ± 2.1	14.6 ± 1.7
F	10.8 ± 3.4	7.6 ± 2.3	9.6 ± 1.5	14.0 ± 2.1
BMI, kg/m^2^	27.2 ± 4.8	25.2 ± 3.6	26.3 ± 4.0	31.1 ± 4.8
M	27.5 ± 4.8	26.1 ± 3.9	27.1 ± 4.1	32.7 ± 5.9
F	27.0 ± 4.8	23.9 ± 2.7	25.4 ± 3.8	30.6 ± 4.3
BMISDS	1.99 ± 0.59	1.95 ± 0.51	1.81 ± 0.53	2.26 ± 0.65
M	1.99 ± 0.57	1.98 ± 0.44	1.82 ± 0.55	2.40 ± 0.78
F	1.99 ± 0.60	1.90 ± 0.61	1.78 ± 0.50	2.21 ± 0.60
WC, cm	89.1 ± 13.2	82.5 ± 11.5	88.5 ± 11.1	100.1 ± 11.7
M	89.9 ± 13.2	84.8 ± 11.8	91.4 ± 11.2	104.4 ± 11.6
F	88.4 ± 13.3	79.0 ± 9.1	85.2 ± 10.2	98.7 ± 11.5
SBP, mmHg	127.1 ± 17.1	121.1 ± 12.6	125.6 ± 14.2	137.3 ± 21.1
M	128.7 ± 16.8	122.0 ± 13.1	129.2 ± 13.9	148.4 ± 19.0
F	125.6 ± 17.3	119.7 ± 11.8	121.4 ± 13.5	133.3 ± 20.4
DBP, mmHg	82.3 ± 10.9	78.7 ± 9.6	81.6 ± 10.6	88.0 ± 10.6
M	83.6 ± 10.8	80.1 ± 9.7	84.1 ± 10.3	93.4 ± 9.4
F	81.0 ± 10.8	76.4 ± 9.1	78.8 ± 10.3	86.0 ± 10.4
SBP percentile	90.4 ± 12.5	89.3 ± 12.2	89.7 ± 14.3	93.0 ± 10.1
M	91.0 ± 12.2	88.9 ± 12.5	91.6 ± 12.9	96.0 ± 6.2
F	89.9 ± 12.8	89.9 ± 10.7	87.4 ± 15.5	91.9 ± 11.0
DBP percentile	91.2 ± 11.5	90.5 ± 11.0	90.0 ± 12.9	93.7 ± 9.8
M	91.8 ± 11.4	91.0 ± 11.2	90.9 ± 12.8	96.8 ± 5.0
F	90.6 ± 11.6	89.9 ± 10.7	88.9 ± 13.0	92.6 ± 10.9
HDL, mmol/l	1.08 ± 0.24	1.15 ± 0.25	1.06 ± 0.23	1.00 ± 0.22
M	1.06 ± 0.23	1.13 ± 0.23	1.05 ± 0.22	0.91 ± 0.20
F	1.09 ± 0.25	1.18 ± 0.27	1.08 ± 0.25	1.04 ± 0.22
LDL, mmol/l	2.23 ± 0.59	2.36 ± 0.63	2.14 ± 0.54	2.18 ± 0.56
M	2.23 ± 0.63	2.37 ± 0.68	2.10 ± 0.53	2.16 ± 0.66
F	2.23 ± 0.54	2.35 ± 0.54	2.18 ± 0.55	2.19 ± 0.52
TG, mmol/l	0.87 ± 0.47	0.84 ± 0.46	0.84 ± 0.42	0.96 ± 0.54
M	0.88 ± 0.52	0.86 ± 0.47	0.83 ± 0.34	1.10 ± 0.89
F	0.86 ± 0.42	0.81 ± 0.44	0.85 ± 0.49	0.91 ± 0.32
Glc0', mmol/l	4.80 ± 0.47	4.73 ± 0.51	4.79 ± 0.45	4.91 ± 0.39
M	4.89 ± 0.44	4.86 ± 0.46	4.85 ± 0.41	5.06 ± 0.39
F	4.72 ± 0.48	4.55 ± 0.54	4.72 ± 0.49	4.86 ± 0.38
Glc120', mmol/l	6.05 ± 1.01	5.99 ± 0.90	6.11 ± 0.99	6.04 ± 1.13
M	6.09 ± 0.94	6.02 ± 0.91	6.02 ± 0.98	6.38 ± 0.91
F	6.00 ± 1.08	5.93 ± 0.89	6.25 ± 1.01	5.90 ± 1.18
Ins0', pmol/l	97.3 ± 62.8	80.3 ± 50.3	101.1 ± 61.0	115.7 ± 73.9
M	92.6 ± 50.7	79.0 ± 44.6	100.2 ± 46.4	114.0 ± 66.3
F	101.8 ± 72.4	82.3 ± 58.0	102.2 ± 75.6	116.4 ± 77.0
HOMA-IR	2.9 ± 2.0	2.4 ± 1.6	3.0 ± 2.0	3.5 ± 2.4
M	2.8 ± 1.6	2.4 ± 1.4	3.0 ± 1.5	3.6 ± 2.2
F	3.0 ± 2.3	2.4 ± 1.8	3.0 ± 2.4	3.5 ± 2.4
ACTH, pmol/l	5.90 ± 4.70	5.37 ± 3.99	6.26 ± 5.88	6.17 ± 3.86
M	6.53 ± 5.49	5.79 ± 4.39	6.74 ± 5.99	8.20 ± 4.10
F	5.30 ± 3.72	4.73 ± 3.21	5.70 ± 4.40	5.40 ± 3.40
Cortisol, nmol/l	327.9 ± 148.9	311.9 ± 138.2	320.3 ± 161.2	359.4 ± 140.8
M	324.8 ± 146.7	308.1 ± 135.6	327.8 ± 161.0	369.2 ± 137.2
F	330.8 ± 151.3	317.8 ± 143.0	313.6 ± 163.4	355.8 ± 142.8

Data are expressed as mean ± SD. BMI = body mass index; DBP = diastolic blood pressure; F = female; Glc0' = fasting glucose; Glc120' = post-challenge glucose; HDL = high-density lipoprotein; HOMA-IR = homeostatic model assessment insulin resistance; Ins0' = fasting insulin; LDL = low-density lipoprotein; M = male; SBP = systolic blood pressure; TG = triglycerides; WC = waist circumference.

**Table 2:** Distribution of cardiovascular risk stratified for weight and sex.

	All	OW	OB
Subjects	406	97 (24.0%)	309 (76.0%)
M	198	47	151
F	208	50	158
Hypertension (>90° percentile)	339 (83.4%)	71 (17.4%)	268 (66.0%)
M	173	36	137
F	166	35	131
Hypertension (>95° percentile)	274 (67.4%)	49 (12.0%)	225 (55.4%)
M	141	25	116
F	133	24	109
HDL <10° percentile	216 (53.2%)	39 (9.6%)	177 (43.6%)
M	123	23	100
F	93	16	77
TG >90° percentile	91 (22.4%)	15 (3.6%)	76 (18.8%)
M	61	11	50
F	30	4	26
LDL >90° percentile	25 (6.2%)	8 (1.9%)	17 (4.3%)
M	17	5	12
F	8	3	5
Dysglycemia	38 (9.3%)	7 (1.7%)	31 (5.4%)
M	21	4	17
F	17	3	14

Data are expressed as absolute values or percentages. HDL = high-density lipoprotein; LDL = low-density lipoprotein; OB = obese subjects; OW = overweight subjects; TG = triglycerides.

### Associations between ACTH, cortisol and metabolic parameters

In the unadjusted analyses, ACTH and cortisol levels were positively associated with SBP, DBP, triglycerides, fasting glucose and HOMA-IR. ACTH, but not cortisol, was positively associated with a higher BMI and insulin levels. Cortisol, but not ACTH, was positively associated with LDL-cholesterol levels (Table 3 and Additional file 2). Adjustment for confounding factors did not change any association for ACTH and revealed a further association with 2-h post-OGTT glucose. However, the association between cortisol and HOMA-IR was lost after adjustment (Table 3).

**Table 3:** Partial correlation for adrenocorticotropic hormone (ACTH) (pmol/l) and cortisol (nmol/l) with cardiovascular risk factors.

Model and factor	ACTH		Cortisol	
	
	r	*P *value	r	*P *value
Model 1				
SBP, mmHg	0.098	0.052	**0.121**	**<0.01**
DBP, mmHg	**0.105**	**<0.03**	**0.119**	**<0.01**
HDL, mmol/l	-0.016	0.753	0.040	0.432
TG, mmol/l	**0.144**	**<0.004**	**0.107**	**<0.03**
LDL, mmol/l	-0.002	0.961	**0.123**	**<0.01**
Glc0', mmol/l	**0.248**	**<0.0001**	**0.110**	**<0.02**
Glc120', mmol/l	**0.144**	**<0.02**	-0.020	0.712
Ins0', pmol/l	**0.122**	**<0.01**	0.050	0.327
HOMA-IR	**0.152**	**<0.003**	0.065	0.200
Model 2				
SBP, mmHg	**0.100**	**<0.05**	**0.112**	**<0.03**
DBP, mmHg	**0.106**	**<0.04**	**0.124**	**<0.01**
HDL, mmol/l	-0.013	0.796	0.048	0.353
TG, mmol/l	**0.128**	**<0.01**	**0.102**	**<0.04**
LDL, mmol/l	-0.019	0.712	**0.116**	**<0.02**
Glc0', mmol/l	**0.218**	**<0.0001**	**0.117**	**<0.02**
Glc120', mmol/l	**0.128**	**<0.04**	-0.030	0.638

Values in bold represent significant results. Partial correlation was adjusted for gender, age, Tanner stage, and BMI in model 1 and for gender, age, Tanner stage, BMI, and insulin resistance in model 2. Log transformation was used for skewed variables. DBP = diastolic blood pressure; Glc0' = fasting glucose; Glc120' = post-challenge glucose; HDL = high-density lipoprotein; HOMA-IR = homeostatic model assessment insulin resistance; Ins0' = fasting insulin; LDL = low-density lipoprotein; SDB = systolic blood pressure; TG = triglycerides.

### ACTH and cortisol levels and cardiovascular risk factors

In the unadjusted analyses, ACTH levels were higher in those with triglycerides ≥90th percentile (*P *<0.003) and LDL-cholesterol ≥90th percentile (*P *<0.04). Higher ACTH levels were also observed in those with impaired fasting glucose or glucose tolerance (*P *<0.001) and BP ≥95th percentile (*P *<0.009), but not in those with HDL-cholesterol ≤10th percentile and BP ≥90th percentile. Cortisol levels were higher in individuals with LDL-cholesterol ≥90th percentile (*P *<0.006) and BP ≥95th percentile (*P *<0.02).

In adjusted models, ACTH levels remained higher in those with triglycerides ≥90th percentile (*P *<0.02) and impaired fasting glucose or glucose tolerance (*P *<0.001). Cortisol levels remained high in those with BP ≥95th percentile and LDL-cholesterol ≥90th percentile.

Higher ACTH levels (third tertile >5.92 pmol/l), although within the normal range, increased the odds of hypertension (>95th percentile), higher triglycerides, impaired fasting or post-OGTT glucose tolerance in the univariate analysis. After adjusting for confounding factors, only the odds of higher triglycerides (OR 2.118, 95% CI 1.139 to 3.939), impaired fasting or post-OGTT glucose (OR 2.548, 95% CI 1.003 to 6.475) remained significant. Higher cortisol levels (third tertile, >383.5 nmol/l), although within the normal range, increased the odds of hypertension (>95th percentile; OR 1.593, 95% CI 1.002 to 3.133) and higher LDL-cholesterol (OR 3.546, 95% CI 1.095 to 11.490) in both univariate and multivariate analyses (Table 4).

**Table 4:** Plasma adrenocorticotropic hormone (ACTH) (pmol/l) and cortisol (nmol/l) tertiles and obesity comorbidities in logistic regression.

Factor	Tertile	ACTH			Cortisol		
		
		OR	95% CI	*P *value	OR	95% CI	*P *value
Hypertension	I	1.000			1.000		
	II	1.088	0.661 to 1.821	0.719	1.071	0.646 to 1.778	0.789
	III	**1.907**	**1.107 to 3.286**	**<0.02**	**1.721**	**1.008 to 2.913**	**<0.04**
HDL <10° percentile	I	1.000			1.000		
	II	1.433	0.884 to 2.323	0.145	0.866	0.549 to 1.429	0.619
	III	1.439	0.886 to 2.338	0.141	1.993	0.610 to 1.614	0.976
TG >90° percentile	I	1.000			1.000		
	II	1.182	0.631 to 2.212	0.601	0.981	0.545 to 1.768	0.950
	III	**2.367**	**1.319 to 4.248**	**<0.004**	1.410	0.798 to 1.491	0.237
LDL >90° percentile	I	1.000			1.000		
	II	2.181	0.655 to 7.263	0.204	2.520	0.676 to 7.490	0.186
	III	3.098	0.973 to 9.866	0.056	**3.223**	**1.012 to 10.265**	**<0.04**
Dysglycemia	I	1.000			1.000		
	II	1.659	0.632 to 4.356	0.304	1.161	0.484 to 2.788	0.738
	III	**2.959**	**1.198 to 7.307**	**<0.01**	1.699	0.740 to 3.900	0.211
Hypertension^a^	I	1.000			1.000		
	II	1.033	0.603 to 1.769	0.906	1.042	0.607 to 1.790	0.882
	III	1.668	0.978 to 2.966	0.072	**1.593**	**1.002 to 3.133**	**<0.04**
HDL <10° percentile^a^	I	1.000			1.000		
	II	1.287	0.771 to 2.148	0.335	0.817	0.489 to 1.365	0.440
	III	1.090	0.649 to 1.832	0.745	0.868	0.514 to 1.467	0.598
TG >90° percentile^a^	I	1.000			1.000		
	III	1.050	0.546 to 2.019	0.884	1.075	0.578 to 2.000	0.819
	III	**2.118**	**1.139 to 3.939**	**<0.01**	1.559	0.851 to 2.856	0.150
LDL >90° percentile^a^	I	1.000			1.000		
	II	2.279	0.678 to 7.665	0.183	2.286	0.678 to 7.725	0.183
	III	3.179	0.975 to10.365	0.055	**3.546**	**1.095 to 11.490**	**<0.03**
Dysglycemia^a^	I	1.000			1.000		
	II	1.582	0.593 to 4.217	0.359	1.040	0.421 to 2.570	0.932
	III	**2.548**	**1.003 to 6.475**	**<0.04**	1.516	0.641 to 3.585	0.344

Values in bold represent significant results. Hypertension was considered as >95° percentile. ^a^Adjusted for gender, age, Tanner stage, and BMI. ACTH tertiles were subdivided as: I = <3.54 pmol/l; II = 3.54 to 5.92 pmol/l; III = >5.92 pmol/l. Cortisol tertiles were subdivided as: I = <240.0 nmol/l; II = 240.0 to 383.5 nmol/l; III = >383.5nmol/l. BMI = body mass index; CI = confidence interval; HDL = high-density lipoprotein; LDL = low-density lipoprotein; OR = odds ratio; TG = triglycerides.

## Discussion

A series of studies in adults and a few studies in selected groups of adolescents, have shown alterations in cortisol in individuals with cardiovascular risk factors (for review see [8,9,13]). In adults, abdominal obesity, high triglyceride and low HDL-cholesterol levels, hypertension, hyperglycemia, MetS and chronic stress have all been characterized by hyperactivity of the HPA axis leading to a functional hypercortisolism. It has also been suggested that inhibiting cortisol action could provide a novel approach for these conditions [8].

In the present study, although ACTH and cortisol levels were within the normal ranges, we observed higher ACTH and cortisol levels in obese children and adolescents with specific cardiovascular risk factors. In particular, ACTH levels were higher in those with higher glucose and triglyceride levels, while cortisol levels were higher in those with hypertension and higher LDL-cholesterol, thereby increasing the risk for these metabolic disturbances.

The first aim of our study was to determine whether cortisol and ACTH were associated with cardiovascular risk factors in obese Caucasian children and adolescents. We showed that ACTH and cortisol were directly associated with glucose, triglycerides, and BP independently of sex, age, puberty, BMI and insulin resistance. Moreover, cortisol was also associated with LDL-cholesterol. These data suggest that the link between HPA and comorbidities in obesity is present in very young children and that elevated ACTH and cortisol levels, although within normal ranges, are already associated with cardiovascular risk factors. The data regarding cortisol are in agreement with the study of Weigensberg and coworkers who demonstrated that cortisol is higher in obese Latino youths with MetS, independent of the degree of obesity and insulin sensitivity [12]. Many studies evaluating cortisol in pediatric obesity have demonstrated an association between cortisol and insulin resistance, leading to hypothesis that a relationship between cortisol and metabolic disturbances would be mediated by insulin sensitivity [9,11,13]. However, both our data and those from Latino youths suggest that the relationship is complex and not only due to insulin resistance. It is well known that Hispanic people have a higher prevalence of type 2 diabetes and cardiovascular diseases [20,23-25], thus such a selected sample of subjects could have quite a different phenotype linked to their genetic susceptibility. Despite this, however, our data and that of others have shown that the association with cardiovascular risk factors remains positive in non-specific populations of obese children and adolescents [9,13].

The lack of association between BMI and cortisol was unexpected, particularly because an association was present for ACTH. However, a number of studies have failed to show an association [12,26-28]. Similar to the lack of an association between BMI and plasma cortisol in the obese population, a lack of association between BMI or body fat levels and urinary free cortisol and free cortisone (in 24-h urine) has also been demonstrated in non-obese children [29]. One explanation could be the homogenous population in terms of weight in our and other studies. However, it could be that ACTH is a better biomarker in childhood in relation to obesity and associated cardiovascular risk factors. Accordingly, major glucocorticoid metabolites in 24-h urine samples (reflecting ACTH-driven adrenocortical activity or cortisol secretion) were significantly associated with body fat in non-obese children [29]. The latter findings, together with our cross-sectional study, suggest that adrenocortical activity (driven by ACTH) is related to body composition during growth whether children are lean/normal weight [29] or obese. However, in this context it is important that higher body fat levels do not always imply higher total blood cortisol although ACTH is increased. ACTH can be increased and total circulating cortisol concomitantly reduced [30]. However, also in children reduced cortisol blood levels are not uncommon in case of elevated body fat levels [31,32]. Moreover, our paper and that of Reinehr and Andler [9] indicate that children's total cortisol plasma levels are not necessarily reduced if body fat is higher. However, really elevated cortisol concentrations in obese children appear to emerge only if a marked insulin resistance is also present [9]. Interestingly, we also observed changes in ACTH and cortisol levels at the last years and at the end of puberty. Healthy adults have higher blood cortisol levels than children. Adults also have clearly higher 24-h excretion rates of free cortisol and free cortisone after correction for body surface area than children. However 24-h excretion of free cortisol and free cortisone in healthy children up to an age of about 14 years is constant after body surface area correction [29,33]. We showed in our obese cohort that cortisol rose in Tanner 4 to 5 stage participants, particularly in females. Conversely, ACTH was higher in males with respect to females in the same pubertal stage and at the age of 14.0 to 15.9 years in presence of still unmodified cortisol levels. These data, with respect to what age ACTH and cortisol start to increase, have to be considered in future works on cortisol in obese adolescents.

We also demonstrated that ACTH levels were associated with metabolic alterations in pediatric obesity, and that some associations were stronger with respect to those of cortisol, in particular insulin resistance. The characteristics of this association suggest that higher ACTH levels could better reflect the interplay between obesity and the HPA axis, and that cortisol-binding globulin (CBG) may be important. Higher CBG levels reduce the rate of cortisol clearance, and thus reflect cortisol levels in plasma [34]. In a large population study, CBG levels were negatively correlated with BMI, BP and insulin resistance, perhaps indicating suppression of CBG synthesis, or a CBG gene polymorphism in obesity [35]. Since stress-induced cortisol pulses are elevated in obesity, lower CBG levels may enhance the glucocorticoid action on tissues and also increase the cortisol clearance. Because CBG is costained with ACTH in corticotrophs and is colocalized with vasopressin in the hypothalamus [34], lower CBG levels may result in higher ACTH levels in obesity by regulating the HPA stress response. However, ACTH levels also represent expression of the negative feedback loop formed by corticotropin-releasing hormone and cortisol, which is influenced by genetic differences in the glucocorticoid receptor [36].

The highest tertiles of the normal ranges of ACTH and cortisol levels in this study were associated with an increased risk of higher triglyceride and LDL-cholesterol levels, respectively. The specific associations for ACTH and cortisol were of interest. The association between cortisol and LDL-cholesterol could be a consequence of multifactorial mechanisms, including direct and indirect effects on lipolysis, free fatty acid production and turnover, and very-low-density lipoprotein synthesis and fatty acid accumulation in the liver (for review see [8,37]). The association between ACTH and triglycerides may be secondary to the strong association between ACTH and insulin resistance in our study. Moreover, ACTH has been shown to increase apolipoprotein E levels in humans, a key protein in determining triglyceride metabolism [38]. However, a higher ACTH-driven adrenocortical activity could have consequences for the hepatic fat and triglyceride metabolism likely through a higher hepatic glucocorticoid metabolism [8,37].

Higher ACTH levels, although within the normal range, were also associated with fasting and post-challenge glucose, and ACTH levels were strong predictors of hyperglycemia. These data are in line with findings in obese Latino youths [11,12], suggesting that changes in the HPA in altered glucose conditions are present also in a broader population. The relationship between glucose and cortisol is in line with glucocorticoid effects on hepatic gluconeogenesis, insulin secretion and resistance [8,39]. However, only ACTH increased the risk of high glucose levels. These findings are concordant with the evidence that lower daily cortisol levels and normal CBG concentrations have been shown in childhood obesity as an age-dependent mechanism to prevent type 2 diabetes [32]. Because ACTH-driven adrenocortical activity or cortisol secretion has been shown associated with body fat in childhood, as previously discussed [29], ACTH might also associate with higher glucose levels before overt disease. In agreement with this hypothesis is that none of the subjects in the present study had overt type 2 diabetes, but they had impaired fasting glucose or glucose tolerance. We also found higher cortisol levels, although within the normal range, in subjects with higher BP, reflecting the data in Latino youths [12]. It is interesting to note that when a cut-off was imposed, cortisol levels were significantly higher only with respect to the 95th percentile of BP, whereas there was no significant association with the 90th percentile, which is suggested as pathological in the MetS definition [18], and is prehypertensive according to the NHBPEP Working Group definition [19]. The lack of an association between cortisol and hypertension using the lower BP cut-off suggests that cortisol levels may be increased only in overt disease. HPA alterations are thus likely to be a consequence of obesity comorbidities, as is also suggested by normalization of cortisol levels after weight reduction [9].

There are limitations in the present study. First is the cross-sectional design, in which we could not determine whether slightly higher ACTH and cortisol levels were a consequence rather than a cause of cardiovascular risk factors in pediatric obesity. Longitudinal studies might clarify this aspect. The second limitation was the inability to define the length of exposure to HPA alterations. The third limitation was evaluation of the HPA without the evaluation of urinary free cortisol. It is difficult to collect daily urine samples properly in pediatric cases, in particular in younger children. In fact, urinary samples were incomplete in most of the children who also took the dexamethasone test for exclusion of Cushing syndrome. However, a single morning fasting cortisol measurement has been shown to be associated with chronic stress and metabolic disturbances [40]. The fourth limitation was the lack of precise data on socioeconomic status due to the refusal of many parents. Socioeconomic status has been found to affect chronic stress and cortisol levels and its role needs to be explored further. The fifth limitation was the absence of true body fat measurements through radiological techniques. However, BMI is a good surrogate for body fat in obesity in large epidemiological sample sizes [41]. The final limitation was the lack of a control group. It would, however, be difficult to choose a good control group for our purpose. Our population was followed in a tertiary care center, and a healthy population of schoolchildren would not be completely comparable in terms of chronic stress. Conversely, the strength of the study was the large sample size, the measurement of post-challenge glucose levels, and the evaluation of many confounding factors.

## Conclusions

In summary, we have shown that obese children and adolescents with cardiovascular risk factors have higher ACTH and cortisol levels, although still within the normal range. These findings have led to the hypothesis that the HPA is involved in obesity comorbidities early in life and in a broad population. Higher ACTH levels are specifically associated with higher triglyceride levels and hyperglycemia, whereas higher cortisol levels are specifically associated with hypertension and high LDL-cholesterol levels. These specific associations suggest complex mechanisms between the HPA axis and metabolic impairments in obesity.

## Abbreviations

AAP: American Academy of Pediatrics; ACTH: adrenocorticotropic hormone; BMI: body mass index; BMISDS: BMI standard deviation score; CBG: cortisol binding globulin; DBP: diastolic blood pressure; HDL: high-density lipoprotein; HOMA-IR: homoeostasis model assessment of insulin resistance; HPA: hypothalamic-pituitary-adrenal; LDL: low-density lipoprotein; MetS: metabolic syndrome; NHBPEP: National High Blood Pressure Education Program; NCEP-ATP: National Cholesterol Education Program-Adult Treatment Panel; SBP: systolic blood pressure.

## Competing interests

The authors declare they have no conflicts of interest.

## Authors' contributions

FP, GB and SB designed the research; RR, SP, VA, GG and CB provided physician care, RR and GA validated the data; FP, GEW and SB analyzed the data; FP, GEW and SB wrote the paper; GA and GB critically discussed the paper. All authors read and approved the final manuscript.

## Pre-publication history

The pre-publication history for this paper can be accessed here:

http://www.biomedcentral.com/1741-7015/11/44/prepub

## Supplementary Material

Additional file 1 **STROBE checklist**.Checklist of items that should be included in reports of observational studies. STROBE = 'STrengthening the Reporting of OBservational studies in Epidemiology'. Click here for file

Additional file 2 **Table S1**.Differences in cardiovascular risk factors and metabolic characteristics according to ACTH (pmol/l) and cortisol (nmol/l) tertiles. Click here for file

## References

[B1] Ogden CL Carroll MD Kit BK Flegal KM Prevalence of obesity in the United States, 2009-2010 NCHS Data Brief 2012 82 1 8 22617494

[B2] De Onis M Blossner M Borghi E Global prevalence and trends of overweight and obesity among preschool children Am J Clin Nutr 2010 92 1257 10.3945/ajcn.2010.29786 20861173

[B3] Nadeau KJ Maahs DM Daniels SR Eckel RH Childhood obesity and cardiovascular disease: links and prevention strategies Nat Rev Cardiol 2011 8 513 525 10.1038/nrcardio.2011.86 21670745 PMC4292916

[B4] Whitworth JA Brown MA Kelly JJ Williamson PM Mechanism of cortisol-induced hypertension in humans Steroids 1995 60 76 80 10.1016/0039-128X(94)00033-9 7792821

[B5] Pasquali R Vicennati V Gambineri A Pagotto U Sex-dependent role of glucocorticoids and androgens in the pathophysiology of human obesity Int J Obes (Lond) 2008 32 1764 1779 10.1038/ijo.2008.129 18838976

[B6] Sukhija R Kakar P Mehta V Mehta JL Enhanced 11β-hydroxysteroid dehydrogenase activity, the metabolic syndrome, and systemic hypertension Am J Cardiol 2006 98 544 548 10.1016/j.amjcard.2006.03.028 16893715

[B7] Walker BR Glucocorticoids and cardiovascular disease Eur J Endocrinol 2007 157 545 559 10.1530/EJE-07-0455 17984234

[B8] Anagnostis P Athyros VG Tziomalos K Karagiannis A Mikhailidis DP Clinical review: the pathogenetic role of cortisol in the metabolic syndrome: a hypothesis J Clin Endocrinol Metab 2009 94 2692 2701 10.1210/jc.2009-0370 19470627

[B9] Reinehr T Andler W Cortisol and its relation to insulin resistance before and after weight loss in obese children Horm Res 2004 62 107 112 10.1159/000079841 15256820

[B10] DeSantis AS Adam EK Doane LD Mineka S Zinbarg RE Craske MG Racial/ethnic differences in cortisol diurnal rhythms in a community sample of adolescents J Adolesc Health 2007 41 3 13 10.1016/j.jadohealth.2007.03.006 17577528

[B11] Adam TC Hasson RE Ventura EE Toledo-Corral C Le KA Mahurkar S Lane CJ Weigensberg MJ Goran MI Cortisol is negatively associated with insulin sensitivity in overweight Latino youth J Clin Endocrinol Metab 2010 95 4729 4735 10.1210/jc.2010-0322 20660036 PMC3050109

[B12] Weigensberg MJ Toledo-Corral CM Goran MI Association between the metabolic syndrome and serum cortisol in overweight Latino youth J Clin Endocrinol Metab 2008 93 1372 1378 10.1210/jc.2007-2309 18252788 PMC2291493

[B13] Barat P Gayard-Cros M Andrew R Corcuff JB Jouret B Barthe N Perez P Germain C Tauber M Walker BR Mormede P Duclos M Truncal distribution of fat mass, metabolic profile and hypothalamic-pituitary adrenal axis activity in prepubertal obese children J Pediatr 2007 150 535 539 10.1016/j.jpeds.2007.01.029 17452232

[B14] Iwen KA Senyaman O Schwartz A Drenckhan M Meier B Hadaschik D Klein J Melanocortin crosstalk with adipose functions: ACTH directly induces insulin resistance, promotes a pro-inflammatory adipokine profile and stimulates UCP-1 in adipocytes J Endocrinol 2008 196 465 472 10.1677/JOE-07-0299 18310442

[B15] Cacciari E Milani S Balsamo A Spada E Bona G Cavallo L Cerutti F Gargantini L Greggio N Tonini G Cicognani A Italian cross-sectional growth charts for height, weight and BMI (2 to 20 yr) J Endocrinol Invest 2006 29 581 593 16957405 10.1007/BF03344156

[B16] Nieman LK Biller BM Findling JW Newell-Price J Savage MO Stewart PM Montori VM The diagnosis of Cushing's syndrome: an Endocrine Society Clinical Practice Guideline J Clin Endocrinol Metab 2008 93 1526 1540 10.1210/jc.2008-0125 18334580 PMC2386281

[B17] Prodam F Trovato L Demarchi I Busti A Petri A Moia S Walker GE Aimaretti G Bona G Bellone S Unacylated, acylated ghrelin and obestatin levels are differently inhibited by oral glucose load in pediatric obesity: Association with insulin sensitivity and metabolic alterations eSpen 2011 6 e109 e115

[B18] Cruz ML Goran MI The metabolic syndrome in children and adolescents Curr Diab Rep 2004 4 53 62 10.1007/s11892-004-0012-x 14764281

[B19] National High Blood Pressure Education Program Working Group on High Blood Pressure in Children and Adolescents The fourth report on the diagnosis, evaluation, and treatment of high blood pressure in children and adolescents Pediatrics 2004 114 555 576 15286277

[B20] Cook S Weitzman M Auinger P Nguyen M Dietz WH Prevalence of a metabolic syndrome phenotype in adolescents: findings from the third National Health and Nutrition Examination Survey, 1988-1994 Arch Pediatr Adolesc Med 2003 157 821 827 10.1001/archpedi.157.8.821 12912790

[B21] de Ferranti SD Gauvreau K Ludwig DS Neufeld EJ Newburger JW Rifai N Prevalence of the metabolic syndrome in American adolescents: findings from the Third National Health and Nutrition Examination Survey Circulation 2004 110 2494 2497 10.1161/01.CIR.0000145117.40114.C7 15477412

[B22] Daniels SR Greer FR Lipid screening and cardiovascular health in childhood Pediatrics 2008 122 198 208 10.1542/peds.2008-1349 18596007

[B23] Weiss R Dziura J Burgert TS Tamborlane WV Taksali SE Yeckel CW Allen K Lopes M Savoye M Morrison J Sherwin RS Caprio S Obesity and the metabolic syndrome in children and adolescents N Engl J Med 2004 350 2362 2374 10.1056/NEJMoa031049 15175438

[B24] Duncan GE Li SM Zhou XH Prevalence and trends of a metabolic syndrome phenotype among U.S. Adolescents, 1999-2000 Diabetes Care 2004 27 2438 2443 10.2337/diacare.27.10.2438 15451913

[B25] Tfayli H Arslanian S Pathophysiology of type 2 diabetes mellitus in youth: the evolving chameleon Arq Bras Endocrinol Metabol 2009 53 165 174 19466209 10.1590/s0004-27302009000200008 PMC2846552

[B26] Pasquali R Vicennati V Activity of the hypothalamic-pituitary-adrenal axis in different obesity phenotypes Int J Obes Relat Metab Disord 2000 24 S47 49 10997608

[B27] Rosmond R Dallman MF Björntorp P Stress-related cortisol secretion in men: relationships with abdominal obesity and endocrine, metabolic and hemodynamic abnormalities J Clin Endocrinol Metab 1998 83 1853 1859 10.1210/jc.83.6.1853 9626108

[B28] Knutsson U Dahlgren J Marcus C Rosberg S Brönnegård M Stierna P Albertsson-Wikland K Circadian cortisol rhythms in healthy boys and girls: relationship with age, growth, body composition, and pubertal development J Clin Endocrinol Metab 1998 82 536 540 9024250 10.1210/jcem.82.2.3769

[B29] Dimitriou T Maser-Gluth C Remer T Adrenocortical activity in healthy children is associated with fat mass Am J Clin Nutr 2003 77 731 736 12600869 10.1093/ajcn/77.3.731

[B30] Jessop DS Dallman MF Fleming D Lightman SL Resistance to glucocorticoid feedback in obesity J Clin Endocrinol Metab 2003 86 4109 4114 11549634 10.1210/jcem.86.9.7826

[B31] Chalew SA Lozano RA Armour KM Zadik Z Kowarski AA Reduction of plasma cortisol levels in childhood obesity J Pediatr 1991 119 778 780 10.1016/S0022-3476(05)80302-6 1941386

[B32] Soros A Zadik Z Chalew S Adaptive and maladaptive cortisol responses to pediatric obesity Med Hypotheses 2008 71 394 398 10.1016/j.mehy.2008.04.020 18547740

[B33] Wudy SA Hartmann MF Remer T Sexual dimorphism in cortisol secretion starts after age 10 in healthy children: urinary cortisol metabolite excretion rates during growth Am J Physiol Endocrinol Metab 2007 293 E970 E976 10.1152/ajpendo.00495.2006 17638704

[B34] Gagliardi L Ho JT Torpy DJ Corticosteroid-binding globulin: the clinical significance of altered levels and heritable mutations Mol Cell Endocrinol 2010 316 24 34 10.1016/j.mce.2009.07.015 19643166

[B35] Fernandez-Real JM Pugeat M Grasa M Broch M Vendrell J Brun J Ricart W Serum corticosteroid-binding globulin concentration and insulin resistance syndrome: a population study J Clin Endocrinol Metab 2002 87 4686 4690 10.1210/jc.2001-011843 12364459

[B36] Witchel SF DeFranco DB Mechanisms of disease: regulation of glucocorticoid and receptor levels--impact on the metabolic syndrome Nat Clin Pract Endocrinol Metab 2006 2 621 631 10.1038/ncpendmet0323 17082809

[B37] Arnaldi G Scandali VM Trementino L Cardinaletti M Appolloni G Boscaro M Pathophysiology of dyslipidemia in Cushing's syndrome Neuroendocrinology 2010 92 86 90 10.1159/000314213 20829625

[B38] Berg AL Rafnsson AT Johannsson M Dallongeville J Arnadottir M The effects of adrenocorticotrophic hormone and an equivalent dose of cortisol on the serum concentrations of lipids, lipoproteins, and apolipoproteins Metabolism 2006 55 1083 1087 10.1016/j.metabol.2006.04.001 16839845

[B39] Lambillotte C Gilon P Henquin JC Direct glucocorticoid inhibition of insulin secretion. An in vitro study of dexamethasone effects in mouse islets J Clin Invest 1997 99 414 423 10.1172/JCI119175 9022074 PMC507814

[B40] Pervanidou P Chrousos GP Metabolic consequences of stress during childhood and adolescence Metabolism 2012 61 611 619 10.1016/j.metabol.2011.10.005 22146091

[B41] Freedman DS Wang J Thornton JC Mei Z Sopher AB Pierson RN Jr Dietz WH Horlick M Classification of body fatness by body mass index-for-age categories among children Arch Pediatr Adolesc Med 2009 163 805 811 10.1001/archpediatrics.2009.104 19736333 PMC2846460

