# Supplementary material for: High-end normal adrenocorticotropic hormone and cortisol levels are associated with specific cardiovascular risk factors in pediatric obesity: a cross-sectional study
Source: BMC Med. 2013 Feb 20;11:44. doi: 10.1186/1741-7015-11-44 (PMC3621818; doi:10.1186/1741-7015-11-44)
Supplement: Additional file 1 — STROBE checklist.Checklist of items that should be included in reports of observational studies. STROBE = 'STrengthening the Reporting of OBservational studies in Epidemiology'. [file 1741-7015-11-44-S1.DOCX]

STROBE checklist

**Title and abstract**

*1a. Indicate the study’s design with a commonly used term in the title or the abstract*

This has been done in both subsections. The study was a cross sectional population based study.

*1b. Provide in the abstract an informative and balanced summary of what was done and what was found*

This has been done with explanation of main outcomes and principal results (pag 3-4).

**Introduction**

*2. Background/rationale. Explain the scientific background and rationale for the investigation being reported*

This has been done Anthropometric and biochemical measurements. See pag. 5.

*3. Objectives. State specific objectives, including any prespecified hypotheses*

This has been done. Aims has been subdivided in several points. See last paragraph pag.6.

**Methods**

*4. Study design. Present key elements of study design early in the paper*

This has been done in the subsection “study design and population” (pag. 7)

*5. Setting. Describe the setting, locations, and relevant dates, including periods of recruitment, exposure, follow-up, and data collection*

All these data have been included in the subsection “study design and population” (pag. 7)

*6. Participants. Cross sectional study*—*Give the eligibility criteria, and the sources and methods of selection of participants*

All these data have been included in the subsection “study design and population” Anthropometric and biochemical measurements” (pag. 7-8)

*7. Variables. Clearly define all outcomes, exposures, predictors, potential confounders, and effect modifiers. Give diagnostic criteria, if applicable*

All these data have been included in the subsections “Anthropometric and biochemical measurements” and “Definitions” (pag. 7-9)

*8. Data surce/measurements. For each variable of interest, give sources of data and details of methods of assessment (measurement). Describe comparability of assessment methods if there is more than one group*

All these data have been included in the subsections “Anthropometric and biochemical measurements” and “Definitions” (pag. 7-9)

*9. Describe any efforts to address potential sources of bias*

Possible bias have been described in the “study design and population” (pag. 7) and have been stressed in study limitations (pag 18).

*10. Study size. Explain how the study size was arrived at*

These data have been included in the subsection “study design and population”(pag 7) and in “statistical analysis (pag. 10)

*11. Explain how quantitative variables were handled in the analyses. If applicable, describe which groupings were chosen and why*

How quantitative variable were handled was explained in the subsection “statistical analysis” (pag.10). The choice of subgroups was explained at subsections “study design and population” and “Definitions” (pag 7-9).

*12. Statistical methods (a-e).*

All information have been provided in the subsection “statistical analysis” (pag.10).

**Results**

*13. (a) Report numbers of individuals at each stage of study—eg numbers potentially eligible, examined for eligibility, confirmed eligible, included in the study, completing follow-up, and analysed. (b) Give reasons for non-participation at each stage(c) Consider use of a flow diagram*

All these data have been included in results, in particular in the subsection “Anthropometric and metabolic phenotype of all group” (pag.11) and in Tables (Tab. 1-2). Nobody refused the study. Flow diagram was not necessary because it was an one step cross sectional observational study.

*14. Descriptive data. (a) Give characteristics of study participants (eg demographic, clinical, social) and information on exposures and potential confounders (b) Indicate number of participants with missing data for each variable of interest (c) Cohort study—Summarise follow-up time (eg average and total amount)*

All these data have been included in results, in particular in the subsection “Anthropometric and metabolic phenotype of all group”. Follow-up time was not described because of the cross sectional nature of the study.

15. *Outcome data. Cross sectional study—Report numbers of outcome events or summary measures*

Measure have been summarized in subheadings.

16. *Main results. (a) Report the numbers of individuals at each stage of the study—eg numbers potentially eligible, examined for eligibility, confirmed eligible, included in the study, completing follow-up, and analysed (b) Give reasons for non-participation at each stage (c) Consider use of a flow diagram*

All these data have been included in results and tables. Nobody refused the consent. Flow diagram was not necessary because it was an one step cross sectional observational study. However, each main outcome was inserted in a subheading to assist for interpretation.

17. *Other analyses. Report other analyses done—eg analyses of subgroups and interactions, and sensitivity analyses*

All results have been represented in univariate and multivariate analysis. The type of analysis was also pointed out in Tables.

**Discussion**

18. *Key results. Summarise key results with reference to study objectives*

Key results have been summarised (see pag. 14). Each objective of the study have been discussed in a separate point respecting the order of introduction and results.

19. *Limitations. Discuss limitations of the study, taking into account sources of potential bias or imprecision. Discuss both direction and magnitude of any potential bias*

Limitations have been discussed at pag. 18 in a specific paragraph.

20. *Interpretation. Give a cautious overall interpretation of results considering objectives, limitations, multiplicity of analyses, results from similar studies, and other relevant evidence*

A cautious interpretation considering other studies has been given for each specific aim.

21. *Generalisability. Discuss the generalisability (external validity) of the study results*

Generalisability has been discussed at each specific point.

**Other information**

22. *Funding. Give the source of funding and the role of the funders for the present study and, if applicable, for the original study on which the present article is based*

Funding has been described in a specific point at pag. 20.
