# Supplementary material for: High-end normal adrenocorticotropic hormone and cortisol levels are associated with specific cardiovascular risk factors in pediatric obesity: a cross-sectional study
Source: BMC Med. 2013 Feb 20;11:44. doi: 10.1186/1741-7015-11-44 (PMC3621818; doi:10.1186/1741-7015-11-44)
Supplement: Additional file 2 — Table S1.Differences in cardiovascular risk factors and metabolic characteristics according to ACTH (pmol/l) and cortisol (nmol/l) tertiles. [file 1741-7015-11-44-S2.DOCX]

Table 1 Supplemental. Differences in cardiovascular risk factors and metabolic characteristics according to ACTH (pMol/L) and cortisol (nMol/L) tertiles.

|  | | **ACTH (pMol/L)** | | | | | | | **Cortisol** **(nMol/L)** | | | |
| --- | --- | --- | --- | --- | --- | --- | --- | --- | --- | --- | --- | --- |
|  |  | **I**  **<3.54** | | **II**  **3.54-5.92** | | **III**  **>5.92** | | **p** | **I**  **<240.0** | **II**  **240.0-383.5** | **III**  **>383.5** | **p** |
| **BMI (kg/m^2^)** | 26.6 ± 4.4 | | 26.9 ± 4.8 | | 28.1 ± 5.1 | | <0.02^b^ | | 26.5 ± 4.3 | 27.5 ± 4.9 | 27.8 ± 5.1 | 0.072 |
| **BMISDS (kg/m^2^)** | 1.94 ± 0.59 | | 1.97 ± 0.56 | | 2.04 ± 0.59 | | <0.05^b^ | | 1.92 ± 0.56 | 2.02 ± 0.60 | 2.01 ± 0.59 | 0.308 |
| **WC (cm)** | 87.5 ± 12.8 | | 88.4 ± 12.4 | | 91.5 ± 14.3 | | <0.01^b^ | | 87.2 ± 12.6 | 91.0 ± 13.3 | 89.2 ± 14.0 | 0.205 |
| **SBP (mmHg)** | 123.6 ± 13.8 | | 125.3 ± 18.0 | | 132.4 ± 17.8 | | <0.0001^a,b^ | | 123.7 ± 16.9 | 126.7 ± 15.6 | 131.1 ± 18.3 | <0.009^b^ |
| **DBP (mmHg)** | 80.5 ± 10.1 | | 81.9 ± 11.2 | | 84.6 ± 11.0 | | <0.008^b^ | | 80.6 ± 10.7 | 82.0 ± 10.7 | 84.2 ± 11.1 | <0.04^b^ |
| **HDL (mMol/L)** | 1.11 ± 0.24 | | 1.06 ± 0.25 | | 1.06 ± 0.24 | | 0.158 | | 1.07 ± 0.23 | 1.10 ± 0.24 | 1.07 ± 0.25 | 0.472 |
| **TG (mMol/L)** | 0.78 ± 0.34 | | 0.86 ± 0.49 | | 0.98 ± 0.53 | | <0.002^b^ | | 0.80 ± 0.38 | 0.85 ± 0.36 | 0.98 ± 0.62 | <0.01^b^ |
| **LDL (mMol/L)** | 2.21 ± 0.51 | | 2.28 ± 0.56 | | 2.21 ± 0.67 | | 0.543 | | 2.16 ± 0.51 | 2.26 ± 0.56 | 2.29 ± 0.57 | <0.05^b^ |
| **Glc0’ (mMol/L)** | 4.62 ± 0.50 | | 4.82 ± 0.42 | | 4.96 ± 0.42 | | <0.0001^a,b,c^ | | 4.71 ± 0.45 | 4.83 ± 0.45 | 4.87 ± 0.48 | <0.01^b^ |
| **Glc120’ (mMol/L)** | 5.83 ± 0.89 | | 6.08 ± 1.10 | | 6.18 ± 1.00 | | 0.071 | | 6.02 ± 0.96 | 6.03 ± 1.12 | 6.08 ± 0.97 | 0.905 |
| **Ins0’ (mUI/L)** | 85.8 ± 55.4 | | 90.8 ± 57.2 | | 115.8 ± 71.4 | | <0.0001^a,b^ | | 86.1 ± 54.1 | 99.8 ± 62.4 | 106.3 ± 70.0 | 0.091 |
| **HOMA-IR** | 2.4 ± 1.6 | | 2.8 ± 1.9 | | 3.6 ± 2.2 | | <0.0001^a,b^ | | 2.5 ± 1.7 | 3.0 ± 2.0 | 3.2 ± 2.2 | <0.04^b^ |

Data are expressed as mean ± SD.

Abbreviations. BMI, body mass index. DBP, diastolic blood pressure. Glc0’, fasting glucose. Glc120’, post-challenge glucose. HOMA-IR, homeostatic model assessment insulin resistance. Ins0’, fasting insulin. SBP, systolic blood pressure. TG, triglycerides. WC, waist circumference.

p value represents post hoc comparisons between the three groups by ANOVA (Bonferroni adjustment). Significant comparisons: ^a^ Group 1 vs. Group 2; ^b^ Group 1 vs. Group 3; ^c^ Group 2 vs. Group 3.
